# Developing a novel deep learning—based model for automatic right ventricular parameters assessment on ctpa in pulmonary embolism

**DOI:** 10.1007/s10140-025-02404-8

**Published:** 2025-10-30

**Authors:** Huairong Zhang, Mengzhou Sun, Lina Miao, Fang Li, Xiaojuan Guo, Li Ma, Xiao Sun, Xiaoyun Liang, Li Zhu

**Affiliations:** 1 Deparment of Radiology, General Hospital of Ningxia Medical University, Yinchuan, 750004 China; 2 Institution of Rsearch and Clinical Innovations, Neusoft Medical System Co.,Ltd., Beijing, 100020 China; 3 Deparment of Anesthesiology, General Hospital of Ningxia Medical University, Yinchuan, 750004 China; 4 Deparment of Radiology, Beijing Chaoyang hospital, capital medical university, Beijing, 100020 China; 5 Institute of Research and Clinical Innovations,Neusoft Medical Systems Co.,Ltd., Shanghai, China

**Keywords:** Pulmonary embolism, Computed tomography pulmonary angiography (CTPA), Deep learning model (DL model), Right heart function

## Abstract

**Purpose:**

To develop an automated method for quantifying right ventricular parameters from CTPA in pulmonary embolism (PE)**.**

**Method:**

In this study, a U-Net based deep learning model was employed to perform automatic segmentation of the right ventricular, left ventricular, ascending aorta, and pulmonary arteries on CTPA images of patients with PE. The right ventricular to left ventricular diameter ratio (RV/LV), main pulmonary artery to ascending aorta diameter ratio (PA/AA), and ventricular septal angle (SA) were automatically calculated using a deep learning model based on anatomical structures. Additionally, two senior radiologists manually annotated the three sets of features to serve as the gold standard. Intra-class Correlation Coefficients (ICCs) were calculated to assess the inter-observer reliability of the proposed method by comparing the three sets of automatically derived measurements with the manual annotations.

**Results:**

The calculated ICCs between automatically derived and manually annotated results demonstrated overall high consistency across the three parameters: RV/LV: 0.76 (95% CI: 0.638–0.877); SA: 0.79 (95% CI: 0.602–0.897); PA/AA: 0.77 (95% CI: 0.672–0.885).

**Conclusion:**

This study developed a deep learning approach for automated and accurate quantification of right ventricular dysfunction in pulmonary embolism (PE), which was shown to be highly consistent with manually annotated results. This provides an automated approach for quantifying right ventricular parameters in PE.

**Graphical Abstract:**

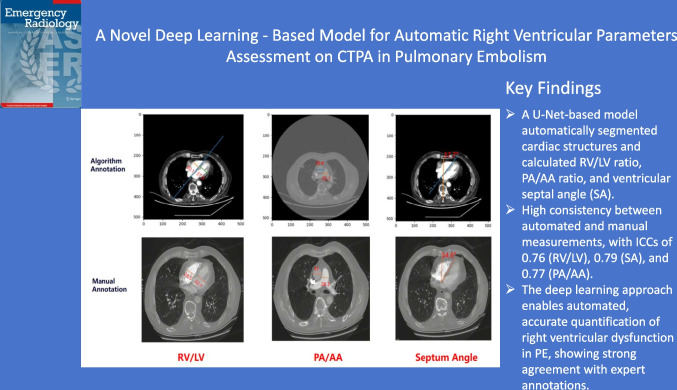

## Introduction

Acute pulmonary embolism (APE) is a severe condition that significantly contributes to cardiovascular mortality and in-hospital deaths [1]. As part of venous thromboembolism (VTE), which includes pulmonary embolism (PE) and deep vein thrombosis, PE patients had the highest in-hospital mortality rate at 7.3% [2]. Computed tomographic pulmonary angiography (CTPA) has emerged as the preferred method for diagnosing APE due to its non-invasion, wide availability, rapid acquisition, and interpretability [3, 4]. This technique has been extensively utilized across emergency departments and hospitals worldwide. However, assessing clot burden and right ventricular dysfunction through CTPA has been correlated with short-term prognosis in acute pulmonary embolism, which may serve as an indicator for guiding treatment decisions [5, 6]. Furlan et al. identified an increased RVd (Right Ventricular Diameter) to LVd (Left Ventricular Diameter) ratio (P = 0.001) as the sole independent factor associated with short-term mortality[7]. Liu et al. showed that the septal angle (SA) and pulmonary artery to ascending aorta diameter (PA/AA) ratio are useful for estimating pulmonary vascular resistance in patients with chronic thromboembolic pulmonary hypertension [8]. Nevertheless, conventional methods for quantifying right ventricular **parameters mainly rely** on manual measurement on CTPA image and they are time-consuming and dependent on the radiologists’ clinical experience, which have not been widely used in clinic practice yet.

Recent advancements in computational technologies and the growing availability of medical data have propelled the integration of deep learning (DL) into medical imaging analysis [9–11]. Significant progress has been made for the detection and identification of PE using deep learning. AI models demonstrate high accuracy in detecting PE on CTPA images, achieving 93% accuracy at the slice—level and 77% at the case – level [12–14]. They can also effectively rule out PE and decrease unnecessary interventions. Furthermore,Liu et al. [10] highlighted the potential of AI-based approaches for evaluating clot burden on CTPA images. Qiao et al. [15] showed that the VB-Net DL model based on CTPA could conveniently and efficiently detect and quantitatively evaluate PE, whereas this study only automatically measure the ratio of RV/LV. Notably, the consistency between these automated assessments and manual measurements performed by radiologists has been rarely assessed as of yet.

To validate concordance between the automated and manual right ventricular function parameters, we developed a U-Net algorithm that directly extracts three key indices from CTPA in acute pulmonary embolism, including right ventricular-to-left ventricular diameter ratio (RV/LV), pulmonary artery-to-aorta ratio (PA/AA), and septal angle (SA). We prospectively evaluated its diagnostic accuracy and computational efficiency against radiologists' measurements to assess its clinical utility for risk stratification and patient management**.**

## Materials and methods

### Study population

Approval for this retrospective study was granted by the Institutional Review Board of the General Hospital of Ningxia Medical University (KYLL-2025–0960), and the requirement for written informed consent was waived. Between January 2024 and February 2025, a total of 420 patients diagnosed with acute pulmonary embolism (APE) were enrolled. Computed tomography pulmonary angiography (CTPA), the standard imaging modality for diagnosing pulmonary embolism (PE), relies on detecting intravascular filling defects within the pulmonary arterial system. Scanning protocols varied slightly across imaging centers, yet all aimed to achieve adequate opacification of the pulmonary arteries and their branches for diagnostic purposes[21].Moreover, the exclusion criteria included the following: (1) CTPA images with poor quality or severe motion artifact; (2) patients who were clinically diagnosed with chronic pulmonary embolism, pulmonary arterial neoplasm, pulmonary vasculitis, and mediastinal fibrosis.

### Image acquisition

CTPA was performed in the craniocaudal direction with multidetector CT scanners (Revolution 512, GE Healthcare; Philips iCT/256) by using a standard CT pulmonary angiography protocol. The whole chest was craniocaudally scanned from lung apex to the lowest hemidiaphragm during a single breath-hold. Scan parameters were as follows: tube voltage of 100–120 kV, tube current of 100–300 mAs, section thickness of 0.625–1.625 mm, table speed of 39.37 mm/s, gantry rotation time of 0.8 s, and reconstruction increment of 1–1.25 mm. A mechanical injector was used for intravenous bolus injection of iopromide (Ultravist, 370 mg/ml, Bayer Schering Pharma) at a flow rate of 5.0 ml/s. For optimal intraluminal contrast enhancement, the automatic bolus-tracking technique had the region of interest positioned at the level of the main pulmonary artery with a threshold of 100 HU predefined threshold, and a fixed delay of 5 s was employed for data acquisition. A soft tissue reconstruction kernel was used for CT image reconstruction.

### Vessel and heart segmentation

**Fig. 1:**
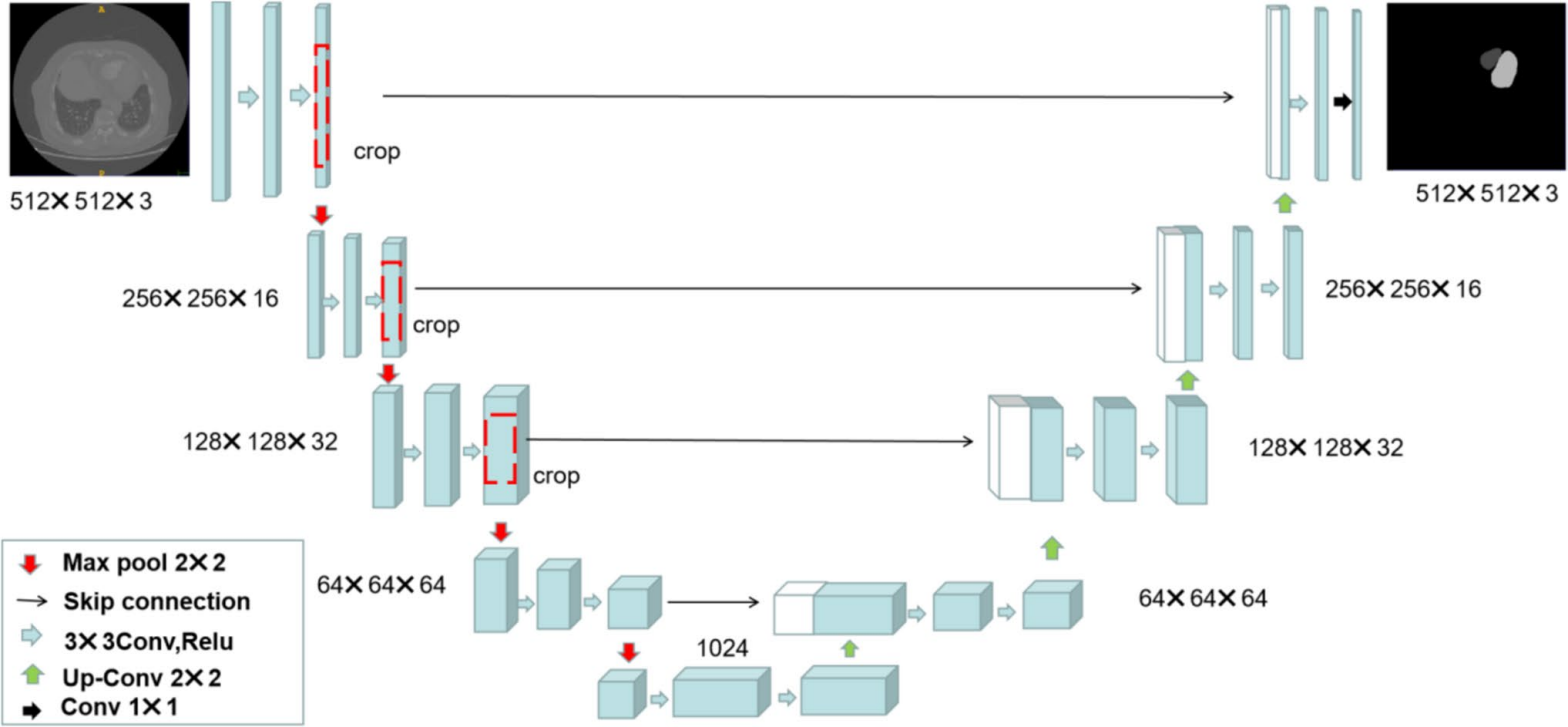
The segmentation results of the left and right ventricles based on the U-Net network. The dark region represents the right ventricle, while the light region corresponds to the left ventricle, clearly delineating the anatomical boundaries and spatial relationship of the ventricles

**Fig. 2:**
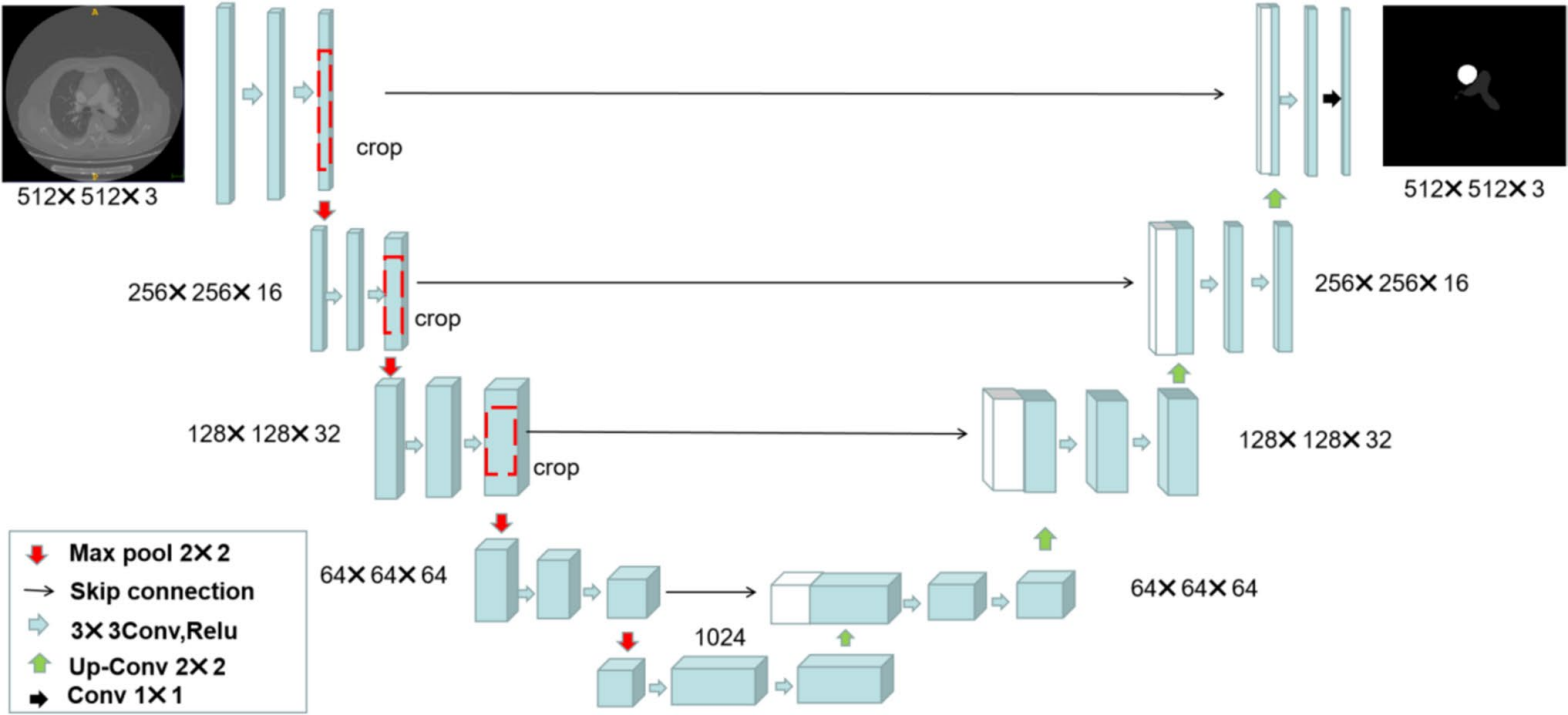
The segmentation results of the pulmonary artery and ascending aorta based on the U-Net network. The dark region represents the pulmonary artery, while the light region corresponds to the ascending aorta, clearly outlining the anatomical boundaries and spatial relationship between the two vessels

In this study, a U-Net segmentation network was implemented using Python 3.8, which was illustrated in Fig. 1 and Fig. 2. U-Net, a widely adopted architecture in medical image segmentation, comprises three main components: an encoder, a decoder, and skip connections. Using CTPA images from patients diagnosed with PE, this model was trained to segment key anatomical structures, including the heart (specifically the left and right ventricle), the pulmonary artery, and the ascending aorta. The vascular annotations were independently performed by two radiologists, each with 5 years of clinical experience. The deep learning model was implemented using PyTorch and trained for 100 epochs with a learning rate set to 0.00001. The input images were preprocessed and resized to a standardized dimension of 512 × 512 × 3 prior to being fed into the model. The optimal checkpoint was selected based on the validation set loss and subsequently evaluated in an independent test set.

### Computation of three sets of clinical indicators

**Fig. 3:**
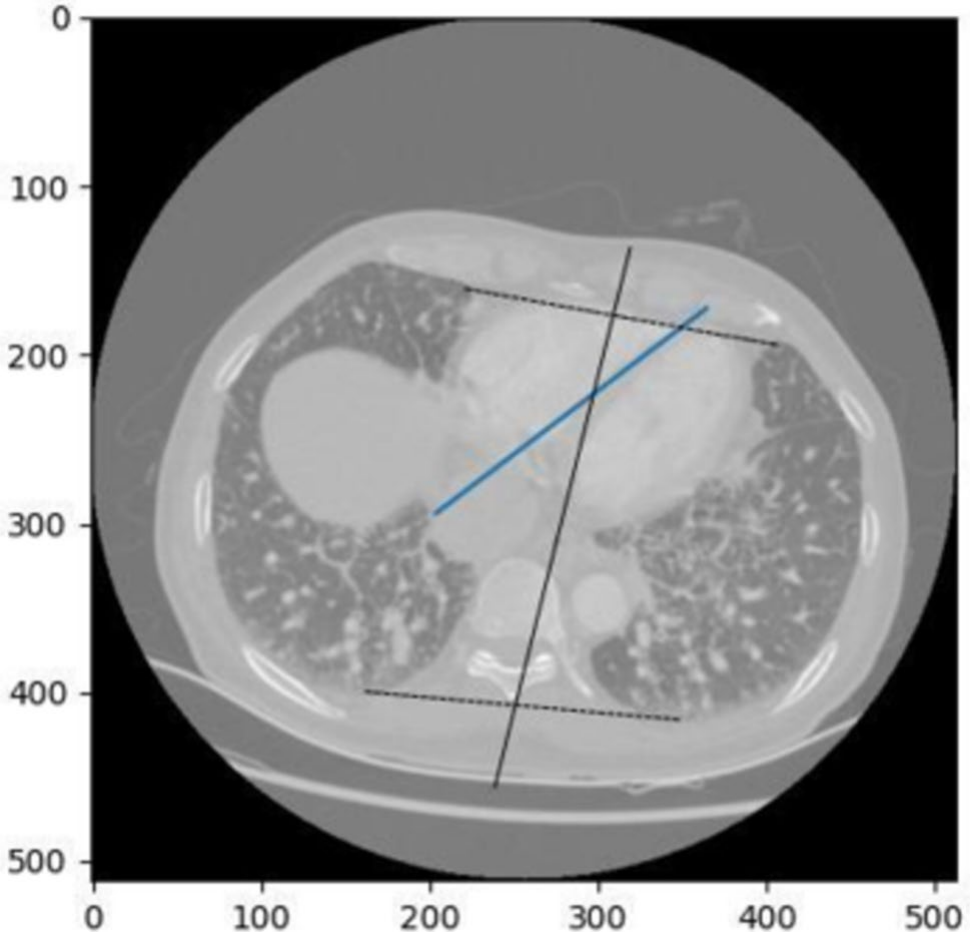
The central black line, which passes through the midpoint of the black line connecting the upper and lower vertices of the lung lobes, represents a line parallel to the spine. The blue line indicates the center line between the two ventricles. The angle formed between these two lines is defined as the ventricular septum angle

The ventricular septum angle refers to the angle formed by the interventricular septum, the wall separating the left and right ventricles of the heart. The sampling plane of the CTPA image was first determined by segmentation mask, i.e., the layer with the largest heart area in the z-axis plane was selected. Based on the existing segmentation mask images from the Unet network, the experiment first identified the closest points between the two cardiac regions and computes the central line between them within the same axial plane. Then, the connecting line between the upper and lower endpoints of the lung segmentation was defined as the line connecting the spine line, as illustrated in Fig. 3. The ventricular septum angle was calculated as the angle between the spinal reference line (black line in Fig. 3) and the midline connecting the centers of the left and right ventricles (blue line in Fig. 3).

**Fig. 4:**
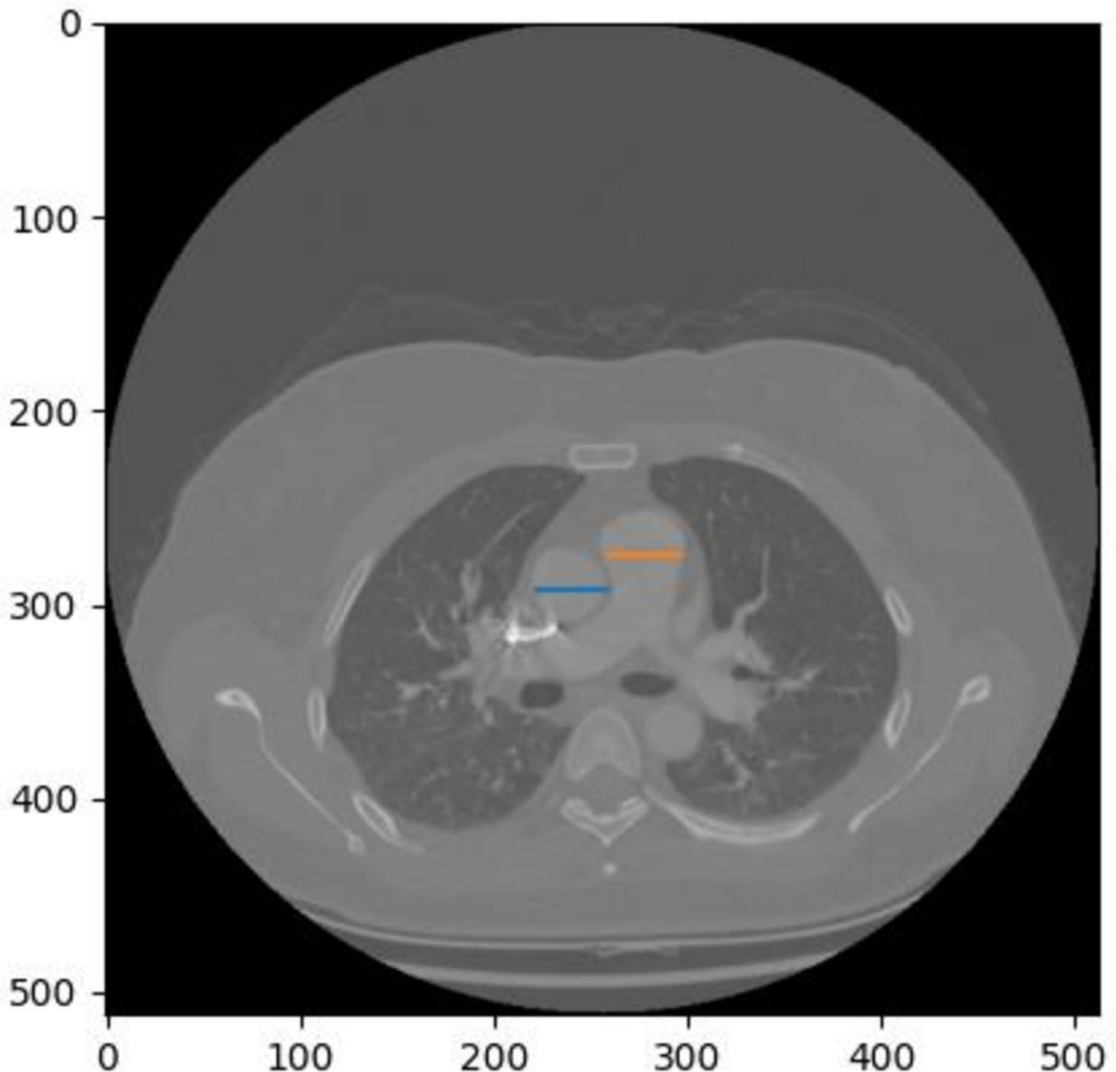
The blue line represented the diameter of the ascending aorta (AA), while the orange line indicated the diameter of the pulmonary artery (PA). The AA/PA ratio was calculated by dividing the length of the blue line by that of the orange line, providing a quantitative measure for comparative vascular assessment

The calculation process for the PA/AA ratio was as follows: First, based on the segmentation masks of the two vessels, the CTPA slice with the largest cross-sectional area of the pulmonary artery was selected as the measurement plane. For the ascending aorta, which appeared as a circular structure on this slice, the maximum diameter was defined as the line connecting the two pixels with the greatest horizontal distance (parallel to the x-axis) within the segmented area—represented by the blue line in Fig. 4. The calculation of the pulmonary artery (PA) diameter followed the same approach as that of the ascending aorta (AA). Within the y-axis range of the segmented PA region, the two points with the greatest horizontal (x-axis) separation were identified, and a line connecting these points was drawn—represented by the orange line in Fig. 4.

**Fig. 5:**
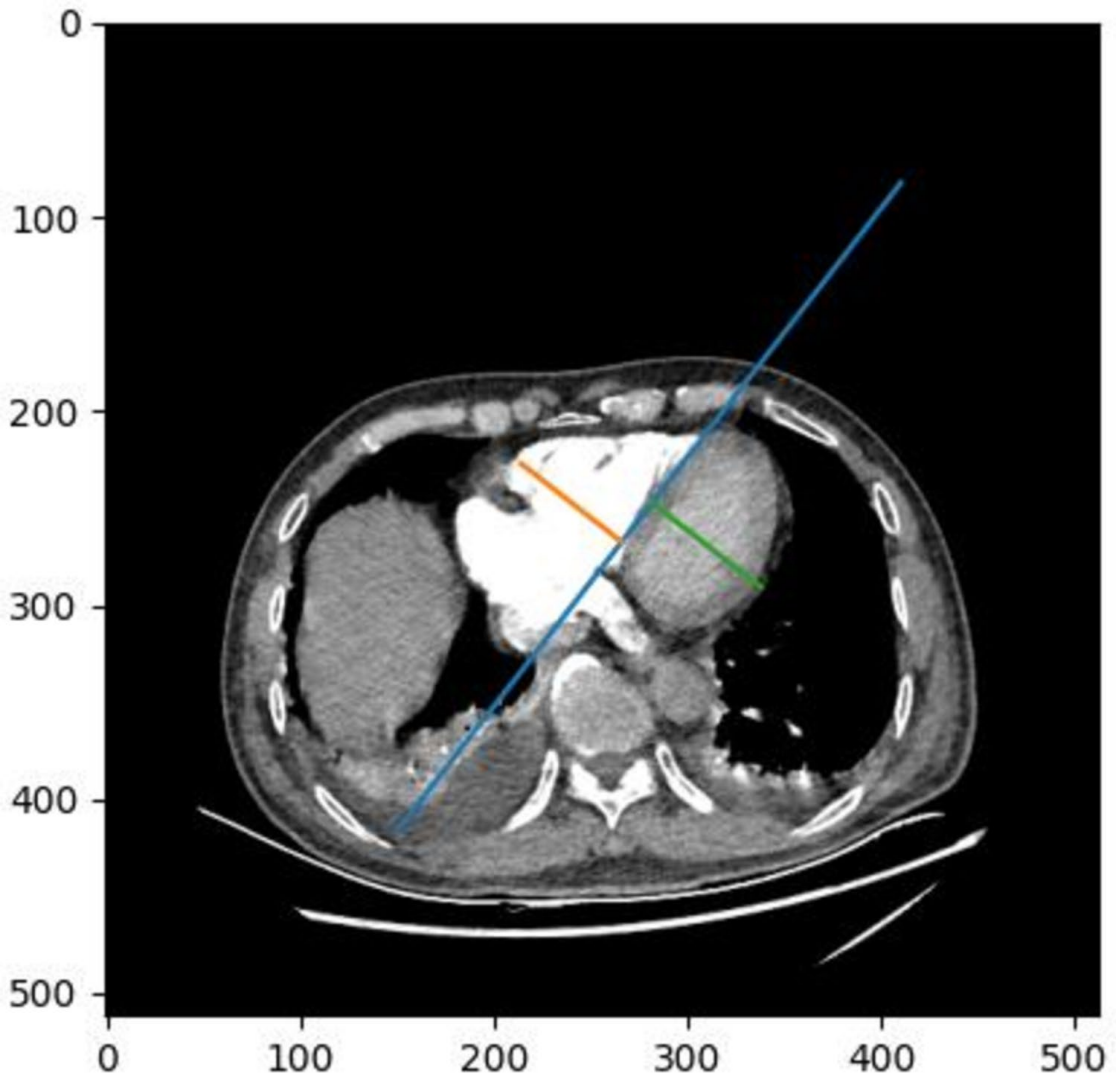
The automatic algorithm marked the right ventricular diameter in orange and the left ventricular diameter in green, clearly delineating the ventricular boundaries for quantitative analysis

The third indicator, the RV/LV ratio, was derived from the segmentation of the right and left ventricles. The algorithm first automatically identified the most representative cross-sectional slice for each patient—specifically, the slice where the combined area of both ventricular cavities was largest and the interventricular septum was clearly visible, consistent with the standard clinical practice[16, 17]. On this selected slice, the maximum diameters of the right ventricle (RV) and left ventricle (LV) were measured, and the RV/LV ratio was calculated by dividing the RV diameter by the LV diameter.The maximum horizontal diameter of each ventricle was calculated by identifying the two points within the segmented ventricular region that were farthest apart along the horizontal axis. The line connecting these two points represented the maximum diameter of the left or right ventricle, respectively. Finally, the two diameters were divided (LV/RV) to derive the clinical index, representing the ratio of the maximum horizontal diameter of the left ventricle to that of the right ventricle (Fig. 5).

The results were primarily evaluated from two perspectives. First, the segmentation performance of the U-Net model was assessed using the Dice coefficient (Formula 1). Second, the accuracy of the three quantitative clinical indicators was evaluated based on Intraclass Correlation Coefficient (ICC) (Formula 2) and measurement accuracy (Formula 3).

Formula 1: $$Dice=\frac{2\left|{X}_{pre}\cap {Y}_{label}\right|}{\left|{X}_{pre}\right|+\left|{Y}_{label}\right|}$$ Formula 2: $$ICC=\frac{{MS}_{R}-{MS}_{w}}{{MS}_{R}+\left(k-1\right){MS}_{w}}$$

Formula 3: $$Acc=\frac{Correct number}{Total number}$$

X_pre_: Prediction results Y_label_: Annotation results MS_R_: between-group mean square.

MS_w_: within-group mean square k: the number of raters.

## Results

### Quantitative assessment

**Fig. 6:**
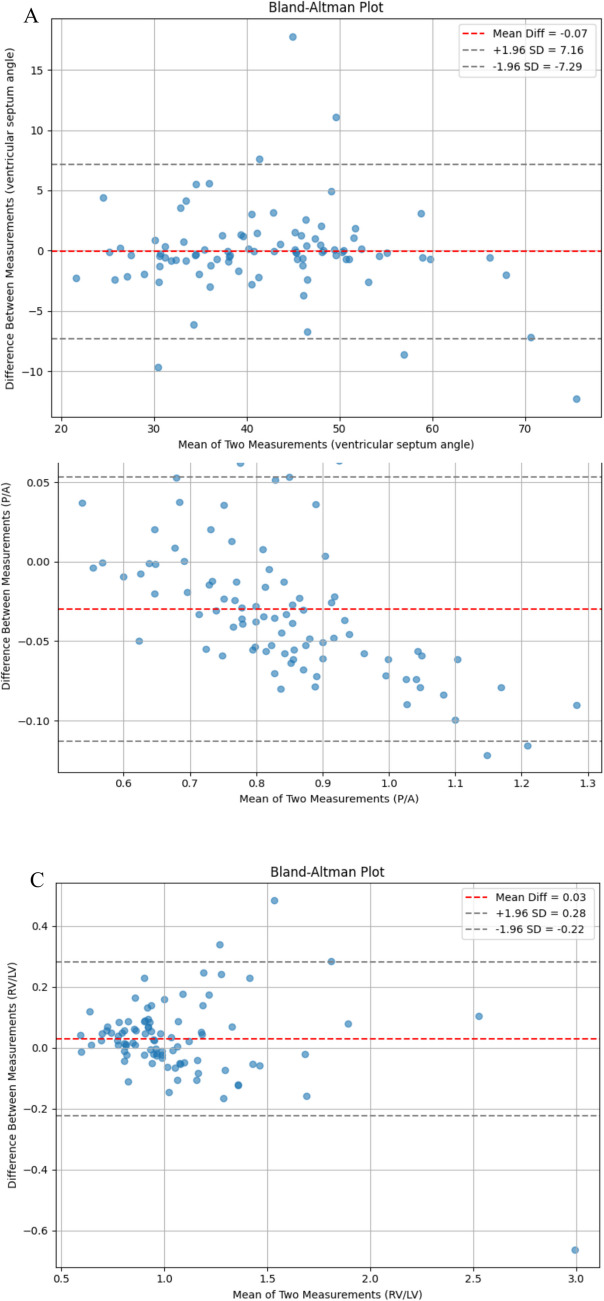
The consistency and deviation between the model and the doctor's measurement results were evaluated by Bland–Altman analysis of three groups (A,SA;B,PA/AA;C, RV/LV)

According to the calculated Dice coefficient, the segmentation results of the four groups of tissues and organs were 0.923, 0.901, 0.887, and 0.891 respectively. The Dice coefficients were calculated by comparing the segmentation outputs of the left ventricle, right ventricle, pulmonary artery, and ascending aorta with the reference annotations provided by experienced radiologists (Table 1). After completing vascular segmentation, three parameters were derived based on the segmentation results: the right-to-left ventricular diameter ratio (RV/LV), the interventricular septum angle (SA), and the pulmonary artery to ascending aorta diameter ratio (PA/AA). These automated measurements were compared against manual annotations, and their agreement was assessed using the intraclass correlation coefficient (Table 2). Based on the calculation of ICC(0.79、0.76 and 0.77), the experiment also conducted Bland–Altman analysis to evaluate the consistency and deviation between the model and the doctor's measurement results (Fig. 6).

**Table 1:** Dice coefficients for the four groups of blood vessel segmentation

Organs	Dice
Left Ventricle(LV)	0.923
Right Ventricle(RV)	0.901
Pulmonary Artery(PA)	0.887
Ascending Aorta(AA)	0.891

**Table 2:** Consistency evaluation of automatically calculated clinical indicators across three patient groups with pulmonary embolism

	ICC	Acc
SA	0.79 (95% cl:0.602,0.897)	0.83 (± 0.03)
RV/LV	0.76 (95%cl: 0.638,0.877)	0.77 (± 0.03)
PA/AA	0.77 (95% cl: 0.672,0.885)	0.79 (± 0.03)

SA:Septum Angle.RV:Right Ventricle.LV:Left Ventricle.PA:Pulmonary Artery.AA:Ascending Aorta.

**Fig. 7:**
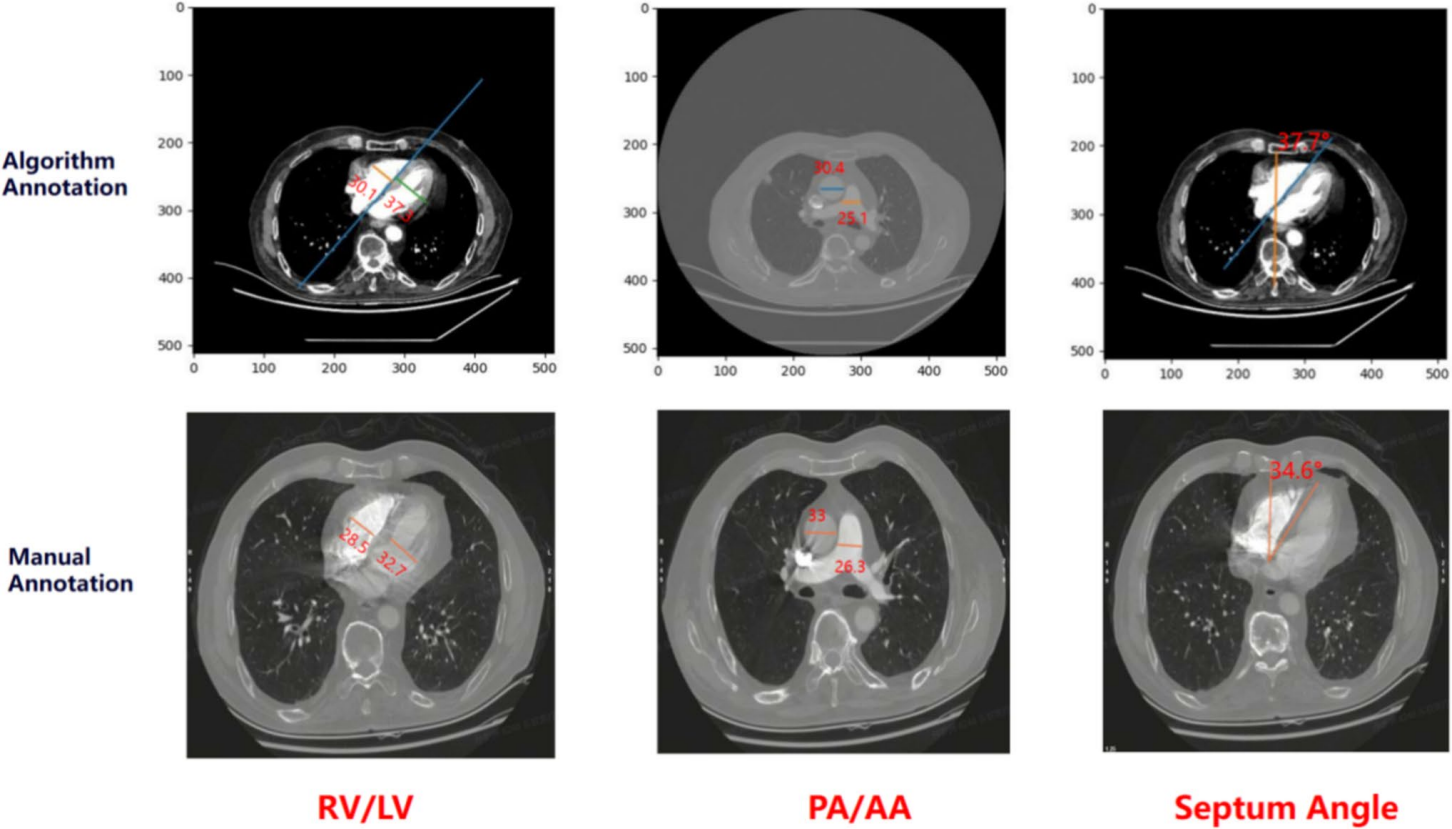
The figure presents a CTPA image from a 72-year-old male patient diagnosed with acute pulmonary embolism following complaints of chest pain and cough. The upper panel shows the annotation results from the experimental algorithm, whereas the lower panel depicts the radiologist’s manual annotations

In addition to the quantitative assessment, a representative case of pulmonary embolism was selected for qualitative analysis, in which the annotations provided by clinical radiologists were compared with the algorithm’s automatically generated results. In Fig. 7, the ratio of RV and LV automatically marked by the algorithm is 1.239, the ratio of RV to LV manually marked by the doctor is 1.147. The ratio of PA and AA automatically marked by the algorithm is 1.211, the ratio of PA to AA manually marked by the doctor is 1.254. The ventricular septum angle was measured as 34.6° manually and 37.7° automatically by the proposed algorithm.

## Discussion

In acute pulmonary embolism (APE), elevated right ventricular afterload stems primarily from mechanical pulmonary vascular occlusion by emboli. This is augmented by vasoconstriction mediated by platelet- and endothelial-derived vasoactive substances (e.g., thromboxane A₂, serotonin)[18, 19]. The resultant acute pulmonary hypertension induces right ventricular (RV) dilatation through pressure–volume overload, increasing stretch on RV myocytes. Consequently, the right ventricular-to-left ventricular diameter ratio (RV/LV) quantified on CTPA directly measures RV dilation—serving as both a biomarker of decompensation and a manifestation of hemodynamic stress[20]. Meanwhile, the main pulmonary artery to ascending aorta diameter ratio (PA/AA) serves as an indirect indicator of pulmonary hypertension, the fundamental cause (increased afterload) leading to RV failure[21]. Furthermore, assessment of septal deviation or flattening provides direct evidence of ventricular interdependence, the pathophysiological mechanism whereby RV failure compromises left ventricular function, creating a detrimental hemodynamic cycle. However, traditional manual measurement methods are time-consuming, subjective, and prone to inter-observer variability[22–24], limiting their widespread applicability and reproducibility.

In this study, we developed a novel deep learning-based model using a U-Net model to automate the segmentation of key anatomical structures on CTPA images and extracted these three critical ventricular parameters. Our image data pipeline was designed with a 512 × 512 × 3 input architecture. First, this configuration enhances computational efficiency under limited hardware resources. Second, it enables simultaneous integration of information across superior and inferior image layers, thereby improving segmentation performance.By integrating anatomical prior knowledge with deep learning techniques, our model achieved satisfactory segmentation accuracy (Dice > 0.85) and strong agreement between automated and manually derived measurements (ICC: 0.74–0.79), demonstrating its reliability and potential for clinical adoption.

Compared with previous studies that focused on limited or incomplete sets of parameters [25, 26], our framework comprehensively computes all three **ventricular parameters**—RV/LV, SA, and PA/AA—enabling more robust risk stratification. For instance, earlier scoring systems such as Qanadli and Mastora scores primarily relied on manual annotation and did not incorporate all three metrics simultaneously [27, 28]. Although Qiao et al. reported automated RV/LV ratio calculations [29], their method lacked validation against manual annotations, potentially limiting its clinical credibility. In contrast, our approach rigorously assessed the quantitative agreement between automated outputs and reference-standard manual measurements (performed by radiologists) across all the three parameter types.

In Delong's [30] experimental study, the RV/LV and PA/AA results of patients with pulmonary embolism were annotated on CTPA images, whereas they were all manually annotated by doctors. Critically, compared with our automatic annotation method, the important indicator of the ventricular septum angle was not considered. It is worth noting that the manual annotations were undoubtedly time-consuming and labor-intensive tasks, which needs to be improved.

There were several limitations in our current study. First, the analysis is based on a relatively modest, single-center cohort. Second, the accuracy of the automated quantitative indicators is contingent upon the precise identification of the corresponding CT slice. Discrepancies between model-selected and manually selected slices may introduce variability into the final parameter measurements. Third, although the algorithm successfully quantified ventricular parameters from CTPA images, its prognostic or diagnostic validity across patient subgroups stratified by right ventricular function was not assessed. Future research should specifically evaluate the algorithm in cohorts with normal versus dysfunctional right ventricles to define clinically relevant diagnostic thresholds for RV/LV ratio, SA, and PA/AA.

## Conclusion

This study presents a transformative deep learning framework for the automated assessment of ventricular parameters in PE patients. By bridging the gap between advanced imaging analysis and clinical decision support, our model offers a faster, more objective, and reproducible solution for evaluating clot burden and right heart dysfunction, thereby enhancing the efficiency and accuracy of PE diagnosis and management in routine clinical practice.

## Data Availability

The datasets used and/or analyzed in the current study are available from the corresponding author upon reasonable request.
